# The French Congress

**Published:** 1889-08

**Authors:** 


### THE FRENCH CONGRESS.

Our contemporary, the *Review*, seems to be considerably worked up over our editorial in the June number on the coming congress to be held in Paris in September, and takes us to task quite severely, and at the same time so inconsistently, as to make it appear that their whole editorial force had contributed to produce the effusion. Dr. DuBois, editor of *L'Odontologie*, however, in noticing our editorial, seems well pleased, and says that "it was an excellent article," and that we "commented favorably upon the letters of MM. Brasseur, DuBois, and Kuhn," and so we intended to do. The *Review* tries to make it appear that we have been dilatory in our action in regard to the congress in that we have not written before on the subject. There are more ways than one of accomplishing things in this world, and we think that if friend Harlan had written less it would have been better for the congress than it is.

As we predicted, the editorial in the May *Review* has raised a storm of opposition that never would have come to the surface had not the second congress matter been broached. Our support of the French congress has been given through its founders, and our journal has really been the official organ of the congress in America, notwithstanding the statement of the *Review* to the contrary. In the March issue of THE INTERNATIONAL appeared the first official announcement of the congress in this country, at least so far as we have seen, in the form of a long letter from Dr. DuBois giving the programme and inviting communications. This was an extra large edition, and copies were sent to all the leading dentists in the United States and Canada. The April number of the JOURNAL contained a letter from Dr. Kuhn, and the June number a long letter from the lamented Dr. Brasseur. The two last letters were written with the especial intention of allaying the fears of Americans regarding the efforts of the *Review* to establish a return congress. It seems strange that the founders of the congress should feel called upon to take steps to be delivered from their friend (?) the *Review*, but such are the facts in the case.

The editorial in question says that "those dentists who are desirous of the establishment of international dental congresses have no animosity towards the dental section of the medical congress." We do not say that they have either, but we do say that so many congresses will tend to divide the interest, and consequently weaken existing organizations. Those of us who are directly connected with our own American Dental Association realize the fact that it is growing harder and harder each year to get suitable material to make a good programme. And why? Simply because of the division of interest on account of the many anniversary meetings of large proportions which have been held in America the past few years.

There are very few writers in the dental profession, and the limit of good matter is not hard to reach; therefore, for the well-being of our already existing institutions, we are opposed to the calling of an international dental congress here in 1892. Let us make a success of what meetings we already have on hand before we strike out into new fields. We will not undertake to discuss the erroneous statement of the *Review* to the effect that "only members of medical societies are admitted to membership in the American Medical Association." The *Review* need only step outside of its door to find the refutation of this statement. Members of the Chicago Dental Club, a flourishing organization numbering between sixty-five and seventy of the most prominent men in the city, presented their certificates as delegates to the American Medical Association and were fully recognized, and took an active part in the deliberations of the Illinois delegation.

Finally, the place to issue the call for an American Congress is in the United States, and not in France. Let the French Congress adjourn *sine die*, and if it is decided to call a dental congress in America in connection with the World's Fair in 1892, let it be done by representatives from all sections of the United States, selected by the different State societies, and not by the self-elected delegates who happen to find it convenient to go abroad this fall. The whole idea is un-American, and we are satisfied will receive a just rebuke at the hands of representative American dentists. What, we would query, makes "it incumbent on the members present at Paris to decide whether it is wise or judicious to hold another International Dental Congress?" If it were absolutely necessary to decide this fall, which it is not, as no other scientific bodies have yet taken any action, then the American and Southern Dental Associations should take it up and appoint joint committees to arrange for the whole matter, subject to the approval of their different organizations next year.

The whole scheme—for by no other name can it be called—is only in keeping with the policy of the *Review* from its inception. Instead of trying to represent the tenor of American thought, it has presumed to dictate to the profession, and its ardent defence of the French Congress has had behind it a long-laid plan to obtain control of a separate dental congress, if one were to be called. But we think the action of the several dental societies that have met since the *Review* unfolded its plan will act somewhat as a check upon its ardor. We predicted that a strong showing of opposition would arise, and so there has, as will be seen from the following resolutions which have been forwarded to us within the past few days.

The Section on Dental and Oral Surgery of the American Medical Association sent a congratulatory telegram to Professor Busch on receiving word that a dental section had been established in the Tenth International Medical Congress, and the general sentiment of all present was undeniably in opposition to calling a separate dental congress.

The Minnesota State Dental Society unanimously passed the following resolutions:

" *Resolved*, That we extend our hearty congratulations to Professor Busch for having secured the establishment of a dental section in the Tenth International Medical Congress, to be held in Berlin, and pledge it our hearty support.

" It is further *resolved*, That we deprecate the calling of a separate international dental congress in America, as has been proposed by the *Review*, on the ground that it will divide the interest and tend to injure the dental section in the Tenth International Medical Congress."

The language is unmistakable, and shows that " especially the Western men," to whom the *Review* appealed, are not responding in the manner desired.

Then the Chicago Dental Club passed strong resolutions, published in the August *Cosmos*, of non-concurrence in the movement, showing that there was anything but harmony in camp over the matter, which would result in the failure of the congress if it were called to meet in Chicago.

The New Jersey State Dental Society, Asbury Park, July 19, while not in favor of passing resolutions pledging support to the Dental Section of the Tenth International Medical Congress, yet, without a dissenting voice, passed the following resolution, taken from the official report of their proceedings, which shows their position as fully as possible on the subject.

The question of appointing delegates to the French Dental Congress being called up, the following gentlemen spoke:

" *Dr. Levy.*—Mr. President, I do not think that we should recognize the French Congress as an international dental congress; therefore I am opposed to appointing delegates to it.

" *Dr. Pinney.*—It seems to me, Mr. President, that we should not recognize the Paris Congress as an international congress at all, and, like Dr. Levy, I object to delegates being sent there."

Dr. Palmer offered the following resolution, which was adopted:

" *Resolved*, that we do not recognize the congress to be held in Paris in September as an international dental congress, and therefore are not in favor of perpetuating it by calling a return congress in America, as proposed by the *Review* in a recent editorial.

"*Dr. Stockton.*—Mr. President, I have noticed in the papers and journals that there are some sixty congresses, already held or to be held, at Paris this year, and I suppose there is no thought that these numerous congresses will be perpetuated and called international congresses hereafter. They will be glad to have you attend, but they have probably no idea whatever that the congress will be perpetuated as an international congress; therefore, as there seems to be no occasion for it, I am opposed to Dr. Palmer's resolution.

"*Dr. Sudduth.*—Mr. President, Dr. Stockton seems not to be conversant with the movement that has been made in Chicago towards perpetuating the congress mentioned in the resolution of Dr. Palmer. Dr. Harlan, in an editorial in a recent number of the *Review*, said very plainly that they proposed to use their utmost endeavors to call a return congress in Chicago, to be held in September, 1892; thereby recognizing the congress to be held in Paris as an international dental congress; and consequently the next congress, proposed to be called at Chicago, will be the Second International Dental Congress.

"*Dr. Palmer.*—Another reason why we should be opposed to the scheme is the fact that steps have been taken recently by the New York Chamber of Commerce towards the celebration of a World's Fair in New York in 1892, and it would hardly be fitting to call our congress at Chicago under those circumstances anyway.

"*Dr. James Truman.*—I have no particular desire to say anything on this question; but I may give an explanation that may perhaps add a little force to the resolution. Three years ago an informal meeting of gentlemen from various parts of the country was held in New York City, at the time of the meeting of the First District Dental Society of New York, for the purpose of organizing an International Dental Association. In view of the fact that the International Medical Congress was to be held the next year, it was postponed, with the expectation that in 1890 we might call an organization of this kind; and it seems to me that when it is called it should be called by delegations from the different dental associations of the world. I am very glad that the resolution was passed.

"*Dr. Peirce.*—Mr. President, I concur in what Dr. Truman has said. My impression is that the Paris Congress is rather a local affair, and not at all international in character; it seems to be part of an exhibition, and that it should be perpetuated, under those circumstances, would seem to be highly improper; and for the reason, also, that in the following year there is to be an international congress at Berlin; and I think it would be unwise for us to divide our forces by having a congress called in this country so near the time of the one to be held in Berlin. I am quite in accord with the resolution.

"*Dr. Waters.*—Mr. President, I do not know that I have anything to say on this question, except to endorse what Dr. Truman has said. I was present at the meeting of the First District Society in New York, when the temporary organization which he spoke of was made. There were gentlemen present from various parts of the country,—the South, the West, and the Northwest,—and this question of holding an international dental congress in the United States was thoroughly discussed. Dr. Northrop, of New York, spoke strongly in favor of the organization, and made an able speech. I think the resolution offered by Dr. Palmer is exceedingly appropriate to the occasion, and I heartily second it and move its adoption.

"Unanimously adopted."

The Pennsylvania State Dental Society, Cresson Springs, July 31, passed the following resolution:

"WHEREAS, The dental meeting to hold session in Paris, known as the French Congress, is not to be deemed a representative international congress; therefore

"*Resolved*, That the Pennsylvania State Dental Society does not recognize the French Congress as in any sense an international congress, and that it declines to appoint delegates to it."

Several of the different journals, following our lead, have conained editorials upon the subject. The *Cosmos*, though always conservative, has the following to say:

"We give the prominence of the editorial department to the following correspondence and report because of the importance of the subject discussed, and because, therefore, of the urgent necessity that definite opinions and conclusions shall be reached concerning it, lest the old-time prediction concerning 'a house divided against itself' be verified in the history of the dental profession;"

and publishes the following letter from Dr. Smith, together with the correspondence between the Chicago Dental Society and the Chicago Dental Club:

"CHICAGO, July 9, 1889.

"TO THE EDITOR OF THE DENTAL COSMOS:

"SIR,—The enclosed correspondence, which explains itself, shows that a very considerable part of the dental profession in Chicago are quite unanimously and strongly opposed to the holding of an international dental congress in this city in 1892. To correct any impression to the contrary which may have been spread abroad, it is desired that the action taken by the Chicago Dental Club be placed before the profession.

"Yours truly,

"C. STODDARD SMITH."

Dr. A. E. Baldwin, Chicago, August *Cosmos*, writes from London, on the date of May 30, upon seeing the editorial in the *Review*, unmasking the plans of its editor, and says,—

"It really provokes a smile to see the *Review* enlarge upon the magnanimous waiting for the Washington meeting to have passed before insisting upon an international dental congress, and how it gave warm support as a journal and personally to the meeting at Washington. To one who has been a close reader and even a warm friend of the *Review* the above statement caused surprise."

Dr. W. W. Allport, in several letters to us personally, has also expressed the same sentiment, as also have several other prominent Chicago dentists.

Dr. Catching, of the *Southern Dental Journal*, comes out with a characteristic editorial on the subject as follows:

"We should or would not have written on this subject at all, but for the last sentence in Dr. Harlan's editorial, where he speaks of adjourning the Paris Congress to meet in the United States in 1892. Here comes up the most objectionable feature, and the main one in his mind. We do not oppose a dental congress in Paris during the great exposition, for, as Dr. Harlan says, other societies will hold congresses there. But those congresses do not propose to cut loose from the International Medical Congress and establish themselves as independent congresses; not a bit of it. And as they are not proposing such a thing, they are supported by their respective followers all over the country, and their Paris meetings will be eminently successful. If Dr. Harlan's object was not to perpetuate the Paris Dental Congress as an independent dental congress, thereby attempting to destroy the dental section in the International Medical Congress, which will meet in Berlin in 1890, and in which is established a dental section, we could raise no objection to the Paris meeting. Really, we do not think it is the sense of the getters-up of the Paris Congress that it should be organized and perpetuated as an independent institution. Such a proposition will have the support of but one journal in America,—viz., the *Dental Review*."

The *American Journal of Dental Surgery*, Chicago, has a long review of the situation in Chicago, of which the following is an abstract:

"We can assure the French dentists who have been laboring to establish dentistry upon its correct basis (a department in medicine) that a majority of the most advanced dentists in America do not favor the project of establishing a perpetual 'International Dental Congress,' and unless they put their feet down strongly against the project of 'Chicago dental politicians' (a little coterie of self-constituted and would-be leaders),—against the project of adjourning their congress to Chicago, or to any other place,—many of the very best men in this country as well as in England and Germany, who would otherwise be present, will refrain from attending their meeting."

Dr. Jonathan Taft, editor of *The Dental Register* and president of the Dental Section of the Ninth International Medical Congress, also vigorously opposes the calling of a separate dental congress in America, and so on. Upon the whole, we think that our prediction in the June number regarding the opposition that would result from the proposed movement upon the part of the *Review* has been very freely fulfilled, and that it is hardly necessary to "flock to Paris to smother" the matter of calling a second international dental congress, in Chicago, at least.

